# Survey of attitudes in a Danish public towards reuse of health data

**DOI:** 10.1371/journal.pone.0312558

**Published:** 2024-12-26

**Authors:** Lea Skovgaard, Claus Thorn Ekstrøm, Mette N. Svendsen, Klaus Hoeyer

**Affiliations:** Department of Public Health, University of Copenhagen, Copenhagen K, Denmark; University of KwaZulu-Natal College of Health Sciences, SOUTH AFRICA

## Abstract

Everyday clinical care generates vast amounts of digital data. A broad range of actors are interested in reusing these data for various purposes. Such reuse of health data could support medical research, healthcare planning, technological innovation, and lead to increased financial revenue. Yet, reuse also raises questions about what data subjects think about the use of health data for various different purposes. Based on a survey with 1071 respondents conducted in 2021 in Denmark, this article explores attitudes to health data reuse. Denmark is renowned for its advanced integration of data infrastructures, facilitating data reuse. This is therefore a relevant setting from which to explore public attitudes to reuse, both as authorities around the globe are currently working to facilitate data reuse opportunities, and in the light of the recent agreement on the establishment in 2024 of the European Health Data Space (EHDS) within the European Union (EU). Our study suggests that there are certain forms of health data reuse—namely transnational data sharing, commercial involvement, and use of data as national economic assets—which risk undermining public support for health data reuse. However, some of the purposes that the EHDS is supposed to facilitate are these three controversial purposes. Failure to address these public concerns could well challenge the long-term legitimacy and sustainability of the data infrastructures currently under construction.

## Introduction

In recent decades, digitalization has resulted in the generation of enormous amounts of data about individuals, not least within the healthcare sector [1, 2]. When data are digitally stored, they can be reused for various purposes. Reuse is essential for certain clinical purposes such as for personalized medicine [3], and can benefit medical research [3], planning and administration [4], as well as commercial innovation [5–7]. Several international agencies are urging countries to increase the collection and reuse of health data [3, 8, 9]. The importance of infrastructures which enable quick and easy access to health data was illustrated during the global COVID-19 pandemic [10]. Authorities in many places looked to countries such as Denmark, where data sharing infrastructures enabled prompt clinical epidemiological studies of the effects of contracting the virus [11] as well as the possibility of monitoring the safety of vaccines [12]. The most ambitious ongoing project in this regard is without doubt the European Health Data Space (EHDS), where the EU Commission envisages a shared data infrastructure and shared data standards for healthcare services throughout the Union in order to facilitate reuse [10].

The use of health data for purposes other than treatment, however, also introduces new risks. Scholars have pointed out that reuse of data poses a risk to individuals’ privacy and autonomy if, for instance, pseudonymized data are reidentified [13], or if individuals are unaware of how health data about them are used [14, 15]. It has also been pointed out that reuse of health data might have adverse effects for some groups [16], through profiling [15] or by increasing inequality in healthcare [17, 18]. It is therefore important to explore how data reuse is viewed by the people the data concern. Understanding how data subjects view questions about data reuse is important both to let citizen values inform policy [19, 20] and to secure the social robustness of data sourcing initiatives [21–24]. Public conflicts about the reuse of health data have previously made policymakers terminate database initiatives. In the UK, the care.data scheme was set up to collect health data from general practice and enable reuse, but it was abandoned after public criticism [21, 25]. Similarly, in Denmark a database from general practice used for research purposes had to be deleted after a public row [22–24]. These cases illustrate the need to understand what people think about reuse of data, and what shapes public support of health data reuse. Accordingly, the EHDS initiative necessitates renewed exploration of citizen attitudes.

Since the EHDS, in many ways, seeks to install across the EU what is already in place on a national scale in Denmark [7], we decided to explore through a survey the question of public attitudes towards reuse of health data in Denmark. Renowned as ‘the epidemiologist’s dream’ [26] Denmark has a long history of sourcing health data [27–29]. Denmark is a welfare state with a highly digitized public sector and extensive registries containing health- and socio-economic information about every registered inhabitant [7, 30]. In addition, legislation grants exemption options for using health data for research without consent [31], similar to what is proposed for the EHDS. Therefore, Denmark offers an opportunity to study public attitudes towards reuse of health data for those interested in understanding attitudes in a country where data reuse has become fully entrenched in everyday practices. The questionnaire was distributed in 2021 during the COVID-19 pandemic which brought attention to the potentials and risks of the reuse of health data for new purposes [32].

Literature reviews on attitudes towards reuse of health data report that people are generally willing to share health data for secondary purposes such as research, policy and planning [32–35]. Reviews, however, also point to how the commercial use of data, data security, privacy and fear of surveillance remain sources of concern [32–37]. Interestingly, a pan-European survey study suggests that Danish respondents are more positive towards the sharing of health data for research and express fewer concerns regarding privacy, compared to respondents from other European countries [38]. While a qualitative study from Denmark argues that, in practice, neither clinicians nor patients pay much attention to consent procedures when health data are collected [39], a considerable amount of the literature both worldwide and from Europe on attitudes towards the reuse and sharing of health data focuses on consent for reuse. A discrete choice experiment found that a majority of people from Northern European countries find the sharing of health data acceptable if they are informed prior to the sharing [40]. The study also found that most people considered it important that data sharing is overseen by a committee. A review of studies from England and Ireland about the reuse of health data, a discrete choice experiment in Northern European countries, and a survey from Germany found opt-out to be the preferred model [35, 40, 41], whereas single-country studies from Sweden and Denmark and a discrete choice experiment in European countries suggest that most people want to give informed consent before data are reused [42–44]. The EHDS is set to rely primarily on opt-out [10]. A review study on attitudes of people in the European Union, however, did not find consistency across the studies in relation to whether participants found consent necessary, and when they did, which consent model they preferred [39]. Similarly, a study on a public debate about data reuse and personalized medicine in Denmark, found that consent was central to discussions about the reuse of health data but that participants disagreed about what counts as “sufficient” consent [45]. Accordingly, the existing literature points towards unresolved issues in relation to the commercial use of data, data security, privacy, surveillance and consent procedures, despite the general willingness to share data.

When in the following we explore attitudes in Denmark, we understand an “attitude” as an individual’s predisposition to evaluate an object in either a positive or a negative way. These evaluations are based on beliefs and emotions in relation to a given object—evaluations which the individual has developed from previous exposure to, or experience with, a given topic [46, 47] and can be said to be tied to people’s beliefs about “human life and social order” [48]. When we throughout the manuscript write “health data” we refer to information pertaining to health either 1) generated in contact with the healthcare sector and stored in patients’ electronic health records or in national registries or 2) collected by individuals about, for example, exercise or private health tests.

With this study we contribute empirically to the literature on public attitudes towards the reuse of health data with survey data from Denmark from 2021 and point to challenges regarding transnational data sharing, commercial involvement, the nation state’s motivation to collect data and the use of data as national economic assets.

## Methods

### Questionnaire development

This questionnaire is a part of a larger project using various methods to study public attitudes to data sourcing and data reuse—in particular as these are enacted in relation to personalized medicine [34, 39, 45, 49]. We used insights gained from our qualitative studies exploring how people talk about reuse of health data, to identify a relevant everyday language and to identify themes relevant to explore in a survey. The questionnaire was developed in conversation between all four authors.

The questionnaire was in Danish and focus on reuse of health data relating to *who should be able to access which data under which conditions*. At the beginning of the questionnaire participants are presented with examples of how health data are collected throughout a typical Danish citizen’s life course during contact with the Danish healthcare sector (from conception to death). At the beginning of each section the questionnaire describes the collection and use of health data relevant for the succeeding questions. The questionnaire consists of thirty-three opinion questions and five background questions. At the end of the questionnaire respondents are asked once what they would consider doing to share their own attitudes about the reuse of health data. When participants were recruited, information on education and region of residency was collected by the host of the online panel.

Surveys are not neutral. They enact realities by introducing respondents to specific themes, providing partial information about these themes and formulating questions covering certain aspects of a given topic [50]. In that way, surveys always shape the attitudes encountered among respondents rather than merely “finding” them. Acknowledging this, we formulated attitude questions symmetrically by formulating them positively (“I think it is positive… [*Jeg mener*, *det er godt*”]) including when our previous research and our qualitative pilot-tests had indicated mostly negative attitudes, such as with respect to data sharing with commercial partners. This is a conscious way of working with acquiescence bias [51]. Questions about concerns were all formulated negatively as “I am concerned…[*Jeg er bekymret over*].” All questions regarding attitudes were formulated as statements that respondents could agree/disagree with. For simplicity, all sections followed the same structure starting with questions about the actors who are traditionally the least controversial (public health institutions or public researchers) followed by questions about actors who are traditionally more controversial (e.g. commercial actors). Participants were asked to indicate on a 5-point Likert scale to which degree they agreed or disagreed with the statement. Participants also had the opportunity to answer “Don’t know [*Ved ikke*]” to attitude questions.

The questionnaire was pilot-tested by the authors (n = 5) using a combination of think-aloud and verbal probing techniques [52] to enhance validity. In this pilot test, we explored whether people perceived the questions as we intended. After this round of pilot-testing we revised the questionnaire according to the comments from the respondents to make the technical aspects understandable in everyday language. We used the most common vernacular Danish terms where possible. For example, we used *‘selvbestemmelse’* (right of self determination) rather than the more specialist Danish term ‘*autonomi*’ (autonomy). Here we report the answers using the most common English translation of the vernacular concepts. Voxmeter, the commercial company hosting the online panel, additionally tested the comprehensibility of the final questionnaire in its online format (n = 125). Here, the company explored the proportion answering “Don’t know,” and the comments to each of the section of questions, and also allowed the respondents to comment on the full questionnaire. The company reported that compared to other questionnaires the proportion answering “Don’t know” was low (with an average of approximately 5%). Only two of the 125 respondents of the pilot test assessed the questionnaire negatively. This pilot test did not give rise to any alterations to the questionnaire.

### Data collection

Research based on anonymous survey data does not require ethical approval according to Danish law, which the Danish National Committee on Health Research Ethics has confirmed to the first author. The survey was distributed through a pre-existing online volunteer opt-in panel [53] by the commercial company Voxmeter. The survey procedure is as follows. Panelists give their written consent to be invited to participate in surveys when they sign up for the panel. They are then regularly invited to participate in surveys, and give their consent to respond to each specific survey when they start answering it. If respondents want to withdraw their consent and opt out while answering a survey, they can do so at any time by closing the survey window. Panelists are recruited through a weekly omnibus survey conducted over the phone. At the end of the weekly omnibus survey respondents are invited to join the online panel. At the time of the study the panel consisted of 100,000 people. The panel is run through an incentive structure whereby every time they answer a survey, panelists gain tokens which they can use either for charity, or at the company’s web shop, or to participate in lotteries hosted by the company. The survey data were collected through email by the company from 18 through 31 March 2021. Stratification proportions were chosen to represent the demographics of the Danish population on the parameters ‘gender’, ‘age’ and ‘region.’ The target sample size was 1000. For typical surveys with a binary outcome, 1000 people will ensure a margin of error of at most 3.1 percentage points. In many examples, where there are more than two outcomes, as is the case for the Likert-scale, the margin of error will be substantially smaller (e.g., less than 2 percentage points) when the outcome is not evenly distributed. We find these margins of error acceptable and have therefore chosen 1000 as the sample size.

The questionnaire was sent to a total of 6000 potential respondents. The total number of included responses was 1071. After 1071 people had answered the questionnaire, and Voxmeter had ascertained that the sample was representative according to the chosen parameters (gender, region), the data collection was closed. The remaining 4929 invitees could not respond after that. There is no agreement on how to calculate response rates for web panels [53] as the sample is chosen based on previously decided stratification proportions.

### Data analysis

We explored attitudes towards reuse of health data by using descriptive statistics. For the data analysis, the 5-point Likert scale was merged into a 3-point Likert scale by merging “Strongly agree” and “Agree” into one category, and “Strongly disagree” and “Disagree” into another single category.

## Results

Table 1 shows an overview of the demographics of the respondents. Regarding age, gender/sex and region, the sample is representative according to 2021 data from Statistics Denmark. The sample therefore reflects the overall demography of Denmark on the chosen parameters.

**Table 1:** Demographics.

	Current sample	Statistics Denmark
**Gender/Sex**		
Male	47.8%	49.7%
Female	51.1%	50.3%
Other	0.2%	-
Prefer not to answer	0.7%	-
**Region**		
Capital	30%	31.8%
Zealand	15%	14.4%
Southern Denmark	23%	21%
Central Jutland	21%	22.8%
Northern Jutland	11%	10.1%
**Education**		
Lower secondary	17.7%	24.5%
Upper secondary	9.6%	11%
Vocational education	23.3%	28.7%
Short (no vocational training, semiskilled worker)	7.9%	5.1%
Middle (skilled worker, theoretical training more than three years)	22.9%	17.9%
Long (theoretical training more than five years)	16.7%	11.7%
Other/don’t know or unspecified	1.8%	1.2%

### Attitudes towards reuse and sharing of health data

We asked participants *who they think should have access to non-anonymized medical records* (Table 2). The greatest support was expressed for access to use medical records for case identification for infectious diseases (79.5% strongly agreed/agreed). The lowest support was expressed for using medical records to detect fraud. For public authorities, 46.8% strongly agreed/agreed and 33.3% strongly disagreed/disagreed that public authorities should be able to use medical records to detect fraud. For insurance companies, the percentages were the opposite: 33.3% strongly agreed/agreed that insurances companies should be able to use medical records to detect fraud, and 46.2% strongly disagreed/disagreed.

**Table 2:** Access to and sharing of health data.

	Strongly disagree/Disagree	Neither agree	Strongly agree/agree	Don’t know
		nor disagree		
**Non-anonymized health data**				
I think relevant health professionals should have the option to use health data from my medical record for case identification for infectious diseases, e.g., MRSA, measles, or COVID-19	7.9%	9.3%	79.6%	3.1%
I think medical students who have previously been part of my treatment should be able to access my medical record later to assess the decisions they made	19.3%	14.9%	62.4%	3.4%
I think health professionals should have the option to use health data from my medical record to find the right treatment for other patients	15%	17.6%	62.9%	4.4%
I think public authorities should have the option to use health data from medical records to find out if patients have committed social fraud to receive public funds, i.e. sickness welfare	33.3%	14.8%	46.8%	5%
I think insurance companies should have the option to use health data from medical records to find out if patients have committed insurance fraud to increase insurance compensation	46.2%	15.5%	33.3%	4.9%
**Anonymized health data**				
I think it is positive if public research institutions, i.e. hospitals and universities, have access to health data	6.2%	13.1%	77.8%	3%
I think it is positive if commercial companies developing pharmaceuticals have access to health data	29.1%	25.5%	39%	6.3%
I think it is positive if commercial companies developing health technologies that are used in the healthcare sector, i.e. CT scanners or machines analyzing test results, have access to health data	23.9%	24.8%	44.9%	6.3%
I think it is positive if commercial companies developing health technologies that are not necessarily used in the healthcare sector, i.e. apps or pedometers, have access to health data	60.5%	22%	12.2%	5.2%
I think it is positive if all commercial companies have access to health data	81.3%	10.7%	4.5%	3.5%
**Use and sharing across borders**				
I think it is positive if commercial companies in other EU countries have access to Danish health data on the same basis as companies in Denmark	53.8%	19.4%	19.8%	7%
I think it is positive if commercial companies outside the EU have access to Danish health data on the same basis as companies in Denmark	69.1%	15.8%	9.2%	6%
I would consider sharing data from my medical record with an organization approved by the EU	38.3%	21.5%	27.6%	12.6%
I would consider sharing data I have collected myself, i.e. about exercise or private health tests, with an organization approved by the EU	42.1%	20.4%	23.6%	13.9%

Table 2 shows the distribution of answers on the Likert scale to attitude questions about access to non-anonymized health data, anonymized health data and sharing and use across national borders.

Table 2 additionally shows answers to questions about *who* respondents think *should have access to anonymized health data*. We found that 77.8% strongly agreed/agreed that it would be positive for public research institutions to have access to health data. In contrast, only 39% responded positively to companies developing pharmaceuticals being allowed access, while 44.9% answered positively to access for companies developing technologies for the healthcare sector. To both questions, around a fourth of the participants reported that they neither agreed nor disagreed. 81.3% strongly disagreed/disagreed that it would be positive for all types of companies to have access, whereas 4.5% strongly agreed/agreed with this statement.

We asked how participants viewed use and sharing of health data *across national borders* (Table 2). We also asked whether participants would be prepared to share health data with an organization which was approved by the EU (Table 2). Of the respondents, 19.8% strongly agreed/agreed that it would be positive for commercial companies from the EU to have access to Danish health data on the same basis as companies from Denmark, whereas only 9.2% strongly agreed/agreed that this should be the case for commercial companies outside of the EU. When asked about *whether participants would themselves consider sharing health data with an organization which was approved by the EU*, 27.6% strongly agreed/agreed that they would consider sharing their own medical record, and 23.3% strongly agreed/agreed that they would consider sharing self-collected health data.

Participants were asked about their attitudes towards reuse of different types of health data for *research purposes* (Table 3). For register data, blood- and tissue samples, samples from COVID-19 tests and blood samples from screening of newborns, 70%–79.9% strongly agreed/agreed that it would be positive if the information were reused for research. Reuse of samples from COVID-19 testing for research into COVID-19 received most support (79.9% strongly agreed/agreed), whereas only a little more than half responded positively to samples from COVID-19 testing being used for research not related to COVID-19.

**Table 3:** Types of health data.

	Strongly disagree/disagree	Neither agree	Strongly agree/agree	Don’t know
		nor disagree		
I think health data from registries should be able to be used for research	5.7%	16.4%	70%	7.8%
I think blood and tissue samples collected as a part of diagnostics or treatment should be able to be used for research	6.8%	15.2%	72.2%	5.8%
I think DNA information collected as a part of diagnostics or treatment should be able to be used for research	14.6%	16.7%	60.5%	8.2%
I think saliva samples from COVID-19 testing should be able to be used for research into COVID-19	5.9%	9.8%	79.9%	4.4%
I think saliva samples from COVID-19 testing should be able to be used to research diseases other than COVID-19	14.8%	23.4%	52%	9.8%
I think blood samples from the newborn screening program should be able to be used for research	7.7%	15.8%	70%	6.4%

Table 3 shows the distribution of answers on the Likert scale to attitude questions about reuse of different types of health data.

### Attitudes towards existing regulation, concerns and fairness of reuse

After a short introduction to the existing regulations on reuse of health data for research in Denmark, participants were asked a set of questions relating to their *satisfaction with the existing rules* (Table 4). To the questions about existing regulations, 25%–33% of respondents answered “Neither agree nor disagree.” 38.7% of respondents agreed that people should have more autonomy in relation to reuse of health data. At the same time, 51.4% of respondents answered that they strongly agreed or agreed that they were satisfied with existing regulation.

**Table 4:** Regulation, concerns and fairness.

	Strongly disagree/disagree	Neither agree	Strongly agree/agree	Don’t know
		nor disagree		
**Regulation**				
I think patients should have more autonomy regarding whether health data about them are used for research	31.8%	25%	38.7%	4.4%
I think the authorities need to ensure that the reuse of health data for research is more tightly regulated	26.4%	33.1%	32.7%	7.8%
I am satisfied with the existing regulation of reuse of health data for research	8.3%	26.2%	51.4%	14%
**Concerns**				
I am concerned about whether the authorities are able to store health data in a secure way	25.1%	23.1%	48.6%	3.3%
I am concerned about Danish welfare state institutions using health data for surveillance	45.2%	22.8%	28%	4%
I am concerned about whether health data are used to discriminate against some citizens	44.6%	21.8%	28.2%	5.3%
I am concerned about whether commercial companies’ interest in financial profit determines how they use health data	11.8%	18.4%	63.2%	6.6%
I am concerned about whether an interest in economic growth determines how health data are used by Danish welfare state institutions	24.6%	26.3%	40.7%	8.4%
I am concerned about whether health data are used outside of Denmark	18.6%	24.6%	50.9%	5.9%
**Fair use**				
I think patients who have received treatment in the public healthcare sector ought to be obliged to share health data which can be used for public research	34.2%	21.8%	37.4%	6.5%
I think patients who have received treatment in the public healthcare sector ought to be able to opt out of health data about them being used for purposes other than their treatment	19.0%	19.3%	55.3%	6.4%
I think it is fair if commercial companies using health data from Denmark profit financially based on their research	32.6%	31.4%	25.4%	10.6%
I think commercial companies using health data from registries should share a part of their financial profit with the state	9.4%	23.2%	56.5%	10.9%

Table 4 shows the distribution of answers on the Likert scale to attitude questions about existing regulation, concerns and fairness of reuse.

We asked whether respondents disagreed or agreed with several statements addressing *concerns in relation to the sharing of health data*, to which the answers can be seen in Table 4. We found the highest levels of concern in relation to profit (i.e. financial profit) based on data (63.2% strongly agreed/agreed with the statement), data sharing outside of Denmark (50.9% strongly agreed/agreed with the statement) and data security (48.6% strongly agreed/agreed with the statement).

Participants were asked about what they thought is *fair* in relation to health data use. More than 50% of the respondents strongly agreed/agreed that patients should be able to opt out of health data about them being used for purposes other than their own treatment. To questions about *distribution of profit* approximately a third of respondents strongly disagreed/disagreed that it is fair for commercial companies to profit (i.e. financially) from research on health data, 31.4% stated that they neither agreed nor disagreed, whereas 25.4% reported that they strongly agreed/agreed that it is fair if companies profit. However, if companies do profit, 56.5% respondents strongly agreed/agreed that the companies should share their earnings with the state (i.e. Denmark).

## Discussion

In this study, we have employed a survey to explore attitudes towards the reuse of health data in Denmark. These findings give indications of the concerns which would be relevant for policy makers to address in order to enhance public legitimacy.

Like authors of other studies, we find that a majority of the respondents are positive towards public researchers being allowed access to health data [33, 35, 42, 43]. However, this proportion declined for answers to questions about health data use by commercial companies or for activities unconnected to the original purpose of the data collection, i.e. for patient care only [37]. Additionally, we found that respondents expressed concerns about companies’ use of data being determined by the companies’ interest in financial profit, and concerns about the Danish welfare state institutions’ interest in health data being based on pursuit of economic growth. These findings are interesting in the light of current Danish and European policies. One objective of the Danish Strategy for Life Science is to ease access to health data, so as to—among other things—attract international companies. Moreover, here the strategy stresses a commercial objective—the likely revenue stream if Denmark succeeds in attracting investors based on potentials of increased use of Danish health data [54]. Likewise, the EHDS aims to enable efficient use of health data for secondary purposes to promote a growth agenda and enhance the health data economy [10].

Literature reviews have suggested that, in some cases, financial profit might be perceived as justifiable when it benefits patients and citizens. For instance, the review by Stockdale et al. describes that, to some, the involvement of private companies is acceptable “when taking into account the possibility of new treatments being discovered (…)” [35]. Similarly, a review by Baines and colleagues suggests that some people are supportive of commercial reuse of health data as long as “any commercial gain that is accrued is secondary to ensuring public benefit” [37]. There is, however, a striking absence of justification of commercial involvement both in Danish policy papers [54] and in the plans for the EHDS [10]. The mismatch between identified preferences and current policies suggests a need in policy making for better justification of commercial access, in order to reduce the risk of citizen protest.

Among the participants in this survey, there also seemed to be a general reluctance towards the sharing of health data across national borders, and a tendency to rate sharing beyond the EU even less positively than sharing with EU countries. Based on our results we cannot determine whether it is the physical location of data or the physical location of the people using data that is causing the concerns expressed in this survey. However, other studies indicate that it matters to some people whether they see themselves as connected to the collective with which they share their data [39]. It might also be a form of lack of identification with the collective that gives rise to the concerns we have found. In Denmark there is no legislation preventing the sharing of Danish health data with researchers in other countries. However, concerns regarding sharing data with researchers outside Denmark is partly addressed by a particular practice at some Danish institutions hosting databases: these specific institutional database hosts insist that, in order to access the data, researchers based in other countries must be associated with a Danish research institution [55]. The reason for this provision is partly to ensure proper understanding of the data. With the EHDS, however, even such national provisions stand to disappear. Our findings suggest this might negatively affect public support.

This study is not the first to identify concerns regarding transnational sharing of data [35]. Yet it is particularly striking to find such strong trends in a study from Denmark with its long history of repurposing health data. It speaks to potential challenges with public legitimacy of initiatives aimed at cross-border sharing, now further facilitated by the legal EHDS framework [10], cross-border data infrastructures such as 1+ Million genomes [56] DARWIN EU [57], and the European Research Infrastructure Consortiums (ERICs) [58]. If large-scale initiatives attract major attention while running counter to citizen expectations and valuations, they might lead to the type of opposition to health data sharing previously experienced in the UK and Denmark [21, 23].

Just over half of the respondents in this study state that they are satisfied with existing regulation around the reuse of health data for research. At the same time, approximately a third of the respondents expressed a wish for stricter regulation and more autonomy for the patients. It should be noted that there is a risk of bias toward assessing the regulation positively in this study as the questionnaire did not ask whether respondents find the current regulation too strict. Other studies have found 50%–60% of citizens expressing a wish to give consent when health data about them are to be used for other purposes [42, 43]. Consent procedures have been criticized for being treated as the general solution to ethical issues in a way that leaves all responsibility with the individual, while topics such as commercial profit and benefit sharing are left unaddressed [59, 60]. We acknowledge these unfortunate implications of a strict focus on informed consent and we are aware that people often consent for reasons other than the information provided [61, 62]. In the current study, people also have conflicting positions on this topic: 31.8% of the respondents of this study disagree with the need for patients to have greater autonomy over how data about them are used, 19% disagree that patients should have the right to opt out of data reuse and 26.4% disagree with the need for tighter regulation. We also acknowledge that our study did not ask participants directly about their attitudes towards consent. Yet our results show that some people express reluctance towards others deciding on data reuse on their behalf. These results are also highly relevant in relation to EHDS, which is set to rely on opt-out. Opt-out allows for some form of autonomy, but only if people are already aware that they have this option, and only if they wish to opt out of all data uses [63]. Our findings, along with previous findings [37], suggest that people hold very diverse attitudes toward different data users (private vs. public entities, local vs. transnational use of data). Still, a majority generally support use of health data for public research, and opt-out of all uses would therefore appeal only to a few data subjects. Accordingly, it might be important to design opt-out solutions with inspiration from tools that allow some form of differentiation, such as meta-consent or dynamic consent [34] when implementing the EHDS, and thereby allow data subjects to opt out of the use of data by specific actors or for specific purposes. Another way of interpreting the wish for stricter regulation and more autonomy for the patients, could be that when authorities decide to overrule patient autonomy, they need to present very weighty reasons for doing so.

Finally, our results show that around 28% express concern that public institutions in Denmark will use data for surveillance or to discriminate against citizens, while around 45% disagree that they are concerned about such misuse of data. Previous studies about attitudes towards biobanking and genetic research from Canada, the USA, the UK and Australia similarly report concerns regarding the possibility of governments accessing and misusing health data [64–66]. We are not arguing that concerns regarding governmental institutions accessing health data are necessarily general barriers to developing data infrastructures for the reuse of health data [37]. Rather, we believe that it is important to understand such concerns in the *context of the study*. In a country like Denmark—where people live their life in close interaction with the welfare state institutions [67] and where the exchange of data between state and citizen is entrenched (and normalized) in the everyday life of citizens [7]—we find 28% to be a substantial minority. This could indicate that the prevalence of concerns related to surveillance and discrimination is likely to be even higher in countries where data reuse is not already integrated into interactions between citizens and state, but installed through EU regulation.

Taken together, these results suggest a need for data guardians to be careful to show themselves trustworthy from the perspective of data subjects. It also clearly indicates a need to install clear limits to the use of health data for objectives beyond health research, and in particular for purposes relating to achievement of goals not related to health (e.g., detection of social fraud and commercial profit), where data might be used against the interests of the data subject. By installing limitations on otherwise laudable goals regarding reuse, it might be possible to safeguard those types of repurposing with highest value for the common good and those most in line with the values of data subjects. The EHDS is taking important steps in this direction, by prohibiting discriminatory use of data and by prohibiting the use of data from the EHDS for the development of products than can harm individuals or societies at large [10]. These steps might prove essential for public legitimacy. For these prohibitions to serve their purpose effectively, they must be thoughtfully implemented, ensuring that individual citizens feel the presence of safety measures.

### Concluding remarks and implications of findings

We found that respondents to this survey in Denmark are generally positive towards the sharing of Danish health data for research conducted by public researchers. However, the further that the reuse of health data is from the original purpose, actors and data-collection context, the more negative are the attitudes that people express. This indicates that the political strategies in Denmark and the European Union to ease access to health data for public and private researchers, and to facilitate the transnational sharing of health data, may well challenge citizen support for the reuse of health data. If the infrastructures facilitating the reuse of health data are to be socially sustainable, we believe that policy makers need to develop ways of regulating commercially oriented research and researchers outside the national context. Our results also point towards a tendency among respondents to want to allow individuals some control over how health data about them are used. The opt-out solution presented in the EHDS regulation will probably not suffice and might work contra to the intended objective by risking a decrease in the quantity of data available in the EHDS. We suggest that for data reuse to find support among these respondents, other forms of expressing autonomy should be considered; and that policies enabling the use of data without consent should thoroughly justify this consent-free use.

## Supporting information

S1 File Survey instrument. (DOCX)
