# Supplementary material for: Survey of attitudes in a Danish public towards reuse of health data
Source: PLoS One. 2024 Dec 26;19(12):e0312558. doi: 10.1371/journal.pone.0312558 (PMC11671018; doi:10.1371/journal.pone.0312558)
Supplement: S1 File — (DOCX) [file pone.0312558.s001.docx]

**S1: Survey instrument (English translation of Danish Survey instrument)**

**Introduction**

In the course of being in contact with the healthcare sector, for instance your general practitioner, at the hospital or when you buy prescription drugs, information about the contact is stored electronically. When you undergo a test using a saliva sample or a blood sample, the sample is sometimes stored. The information and samples are stored to document treatment and sometimes they are also used for other purposes.

This survey is about your attitudes towards **use of health information for purposes other than treatment.**

There are no right or wrong answers. You just have to share your immediate attitude.

**Examples of health information, which are stored about citizens:**

- Results from the 12 week ultrasound undertaken during pregnancy
- Drugs provided to the mother during labor
- Results from test taken from newborns
- Vaccination
- Information about contact to the general practitioner, for instance prescribed medicine
- Information about contact to a hospital, for instance reason and duration of admission
- Information about rehabilitation, for instance type and duration
- Course of death

Throughout the survey, there will be texts explaining how health information is currently being used. After each section, you will have the possibility to comment on the questions you answered in the preceding section.

1. **Questions about who you think should be allowed to access information from patients’ medical records**

Sometimes, patients’ medical records are used for other purposes than treatment. We would like to know **who** you think should be able to access information from patients’ medical records.

To what extent do you agree or disagree with the following statements?

Response categories:

Strongly disagree

Disagree

Neither disagree or agree

Agree

Strongly agree

Don’t know

- I think relevant health professionals should have the option to use health information from my medical record for case identification for infectious diseases, e.g., MRSA, measles, or COVID-19.
- I think medical students who have previously been part of my treatment should be able to access my medical record later to assess the decisions they made.
- I think health professionals should have the option to use health information from my medical record to find the right treatment for other patients.
- I think public authorities should have the option to use health information from medical records to find out if patients have committed social fraud to receive public funds, i.e., sickness welfare.
- I think insurance companies should have the option to use health information from medical records to find out if patients have committed insurance fraud to increase insurance compensation.

If you have any comments on the questions posed above, you can write them here.

1. **Questions about how you think access for researchers to anonymous health information should be regulated**

In Denmark there are several **registries that store information about contacts with the healthcare sector.** For instance, there is a register containing information about admissions to hospitals and a register containing information about every patient diagnosed with cancer. Some researchers use information from **registries** in their research. In this types of research it is not important to know who the information is about. Therefore, name, addresses and CPR-numbers are removed, making the information from the register **anonymous.**

In order to access information from Danish registries researchers have to apply for **permission from the authorities.** Projects assessed as furthering the interests of society are granted access.

**The person the information is about** **does not** have to give permission for health information from registries to be used for research. In case the research involves biological material, such as blood, the person must consent to the material being used and the researchers also have to apply for permission from a scientific ethics committee. In some cases, the committee can allow research based biological material without consent.

We want to know whether you think this way of regulating access for researchers **should be changed.**

To what extent do you agree or disagree with the following statements?

Response categories:

Strongly disagree

Disagree

Neither disagree or agree

Agree

Strongly agree

Don’t know

- I think patients should have more autonomy regarding whether health information about them is used for research.
- I think the authorities need to ensure that the reuse of health information for research is more tightly regulated.
- I am satisfied with the existing regulation of reuse of health information for research.

If you have any comments on the questions posed above, you can write them here.

**3. Questions about who you think should be able to apply for access to anonymous health information**

We would like to know who you think should have access to anonymous health information **provided that they have permission from the authorities.**

To what extent do you agree or disagree with the following statements?

Response categories:

Strongly disagree

Disagree

Neither disagree or agree

Agree

Strongly agree

Don’t know

| - I think it is positive if public research institutions, i.e., hospitals and universities, have access to health information. - I think it is positive if commercial companies developing pharmaceuticals have access to health information. - I think it is positive if commercial companies developing health technologies that are used in the healthcare sector, i.e., CT scanners or machines analyzing test results, have access to health information. - I think it is positive if commercial companies developing health technologies that are not necessarily used in the healthcare sector, i.e., apps or pedometers, have access to health information. - I think it is positive if all commercial companies have access to health information.   If you have any comments on the questions posed above, you can write them here. |
| --- |

Think of the companies you have just stated should be granted access. We would like to know if it matters to you whether the companies are based in **Denmark or in other countries.**

To what extent do you agree or disagree with the following statements?

| - I think it is positive if commercial companies in other EU countries have access to Danish health information on the same basis as companies in Denmark. - I think it is positive if commercial companies outside the EU have access to Danish health information on the same basis as companies in Denmark. |
| --- |
| If you have any comments on the questions posed above, you can write them here.  **There** is talk about creating databases where **citizens in the EU can share** information from their medical record and health information they themselves collect. In this way, citizens can themselves decide who can access this information. This could be **doctors in other countries, public researchers or companies** developing health technology in other EU countries.  To what extent do you agree or disagree with the following statements? |
| - I would consider sharing information from my medical record with an organization approved by the EU. - I would consider sharing health information I have collected myself, i.e., about exercise or private health tests, with an organization approved by the EU. |
| If you have any comments on the questions posed above, you can write them here. |

**5. Questions about which types of health information you think it should be possible to use for research**

We would like to know whether there are **some types of anonymous health information** which you think should or should not be used for research **provided that the authorities have given permission to the use.**

To what extent do you agree or disagree with the following statements?

| - I think health information from registries should be able to be used for research. - I think blood and tissues samples collected as a part of diagnostics or treatment should be able to be used for research. - I think DNA-information collected as a part of diagnostics or treatment should be able to be used for research. - I think saliva samples from COVID-19 testing should be able to be used for research into COVID-19. - I think saliva samples from COVID-19 testing should be able to be used to research diseases other than COVID-19.   In Denmark we have a screening program for newborns. The screening is conducted by taking a blood sample from the infants’ heel and testing the blood for a range of congenital diseases. Afterwards, the blood sample is stored and can be used later, for instance for research. |
| --- |
| \|  \| \| --- \|  - I think blood samples from the newborn screening program should be able to be used for research |

If you have any comments on the questions posed above, you can write them here.

**5. Questions about whether you have concerns regarding use of health information**

To what extent do you agree or disagree with the following statements?

- I am concerned about whether the authorities are able to store health information in a secure way.
- I am concerned about Danish welfare state institutions using health information for surveillance.
- I am concerned about whether health information are used to discriminate against some citizens.
- I am concerned about whether commercial companies’ interest in financial profit determines how they use health information.
- I am concerned about whether an interest in economic growth determines how health information are used by Danish welfare state institutions.
- I am concerned about whether health information is used outside of Denmark.

If you have any comments on the questions posed above, you can write them here.

**7. Questions about when you think use of health information is fair**

The next questions are about what you think is **fair** in relation to use of health information.

To what extent do you agree or disagree with the following statements?

- I think patients who have received treatment in the public healthcare sector ought to be obliged to share health information which can be used for public research.
- I think patients who have received treatment in the public healthcare sector ought to be able to opt out of health information about them being used for purposes other than their treatment.
- I think it is fair if commercial companies using health information from Denmark profit based on their research.
- I think commercial companies using health information from registries should share a part of their profit with the state.

If you have any comments on the questions posed above, you can write them here.

**8. Questions about what you could think of doing to share your attitude**

- Could you think of **sharing** your opinion about use of health information in some of the following ways? Please feel free to check multiple options.

Write an opinion piece

Make a social media post, for instance on Facebook, Twitter or Instagram

Share an article or a post that someone else made on social media, for instance on Facebook, Twitter or Instagram

Participate in a research interview if you were invited

None of the above

Don’t know

**9. Background questions about you**

Finally, we would like to ask some questions about your background and your previous contact with the healthcare system.

- What **gender** do you identify as?

Male

Female

Other

Prefer not to answer

- Do you consider yourself to belong to a minority? Please feel free to check multiple options.

Yes, sexual minority

Yes, ethnic minority

Yes, religious minority

Yes, other, please specify

No

Prefer not to answer

- Would you say that you have had a lot of contact with the healthcare system? Please feel free to check multiple options.

Yes, as a patient

Yes, as a relative of a patient

Yes, because I am a trained healthcare professional

Other, please specify

No

Prefer not to answer

- Would you say that you are generally satisfied or dissatisfied with your contact with the healthcare system?

Very satisfied

Mostly satisfied

Neither satisfied nor dissatisfied

Mostly dissatisfied

Very dissatisfied

Don't know

Prefer not to answer
